# Cell Sheet Technology as a Platform for Functional Regeneration

**DOI:** 10.3390/ijms27198695

**Published:** 2026-09-29

**Authors:** Goshi Shiota, Noriko Itaba

**Affiliations:** Division of Regenerative Medicine and Therapeutics, Department of Genomic Medicine and Regenerative Therapy, School of Medicine, Faculty of Medicine, Tottori University, Yonago 683-8503, Tottori, Japan; itaba@tottori-u.ac.jp

**Keywords:** regenerative medicine, tissue engineering, cell sheet technology, functional regeneration, liver fibrosis, liver cirrhosis, anti-fibrotic therapy, paracrine factor, mesenchymal stem/stromal cells, clinical applications

## Abstract

Cell sheet technology has evolved into a unique scaffold-free and biomaterial-free regenerative medicine platform that enables transplantation of intact cellular constructs while preserving extracellular matrix and cell–cell interactions. Clinical applications of cell sheet technology have demonstrated successful tissue and organ regeneration in the cornea, esophagus, heart, cartilage, and retina. More recently, the therapeutic scope of cell sheet technology has expanded beyond tissue replacement toward stem cell-based regenerative therapy, in which paracrine signaling, anti-fibrotic activity, and microenvironment remodeling play important roles. In this review, we use the term “functional regeneration” as an integrative therapeutic framework of a regenerative medicine platform capable of promoting the regenerative processes that modify disease pathophysiology and may alter the natural history of a disease, irrespective of complete anatomical reconstitution. Liver cirrhosis is a representative target disease for this concept of functional regeneration because the functional regenerative therapy that reverses progressive fibrosis and restores hepatic function has the opportunity to prevent the onset of hepatocellular carcinoma. This review discusses the evolution of cell sheet technology, the biological basis of stem cell-based functional regeneration, and the development of KCS-003, a cell sheet-based therapeutic product manufactured from mesenchymal stem/stromal cells preconditioned with IC-2, which has potent anti-fibrotic action and promotes liver regeneration. In addition, clinical investigations, including corneal and retinal reconstitution, esophageal regeneration, cartilage repair, and cardiac patches, were shown as representative clinical cases of cell sheet technology.

## 1. Introduction

Regenerative medicine is an emerging and compelling field of medicine. Regenerative medicine increasingly relies on the use of innovative biomaterials and stem cell-based strategies for tissue repair and regeneration [1,2,3,4,5,6]. Among these approaches, stem cell therapy has emerged as a promising therapeutic option due to the unique ability of stem cells to self-renew and differentiate into multiple cell lineages [7,8,9,10,11,12,13,14,15,16,17]. Human pluripotent stem cells (hPSCs) and mesenchymal stem/stromal cells (MSCs) have emerged as essential components of regenerative medicine owing to their therapeutic potential. Among these, hPSCs are distinguished by their capacity for indefinite self-renewal and their ability to give rise to a broad spectrum of specialized cell types originating from the ectoderm, mesoderm, and endoderm [11]. Mesenchymal stem/stromal cells (MSCs) are multipotent cells capable of differentiating into a variety of cell lineages, a property that underlies their broad applicability in regenerative medicine and clinical practice [12,13,16,17]. The increasing number of clinical trials has generated valuable evidence regarding the safety and therapeutic potential of stem cell-based interventions across a broad range of diseases [11].

Stem cells often require appropriate scaffold systems to maximize their biological functions. Advanced biomaterials, including hydrogels and nanoparticles, can provide a supportive microenvironment for stem cell proliferation and differentiation while also serving as vehicles for the controlled delivery of growth factors and therapeutic agents to enhance tissue regeneration [1,3]. Currently, advances in tissue engineering have been driven by the development of biomimetic scaffolds and the application of stem cell technologies, both of which have significantly improved tissue regeneration for a variety of clinical conditions. The extracellular matrix (ECM) functions as a dynamic microenvironment that regulates cellular activities through complex biomechanical and biochemical signals, thereby playing a central role in tissue repair and regeneration [18]. Inspired by the structure and function of the native ECM, ECM-mimicking biomaterials have emerged as promising platforms for regenerative medicine. In tissue engineering, biomaterials are generally categorized into ECM-derived materials, synthetic polymers, natural biomaterials, bioceramics, and composite materials [18]. Despite the distinct characteristics of each biomaterial class, several common challenges remain. First, achieving adequate immune compatibility continues to be a major concern, as host immune responses can trigger foreign body reactions characterized by chronic inflammation, foreign body giant cell formation, fibrosis, and subsequent tissue damage [19]. Second, toxicity may arise from degradation products or residual chemical crosslinkers. Third, successful integration with host tissues is often hindered by mechanical incompatibility, which may promote fibrotic encapsulation and undesirable tissue remodeling. Finally, the long-term safety of biomaterials has yet to be fully established, particularly with respect to delayed inflammatory complications [18].

A major obstacle in regenerative medicine is the inefficient delivery and engraftment of therapeutic cells. In many cases, only a limited proportion of transplanted cells successfully reach the injured tissue, maintain viability, and participate in tissue repair, underscoring the need for strategies that promote effective cell engraftment [20]. Current stem cell therapies based on single-cell injections often suffer from substantial post-transplantation cell loss, resulting in poor tissue retention and transient therapeutic effects [21]. These observations suggest that the effectiveness of cell therapy is influenced not only by cell type and disease indication but also by the delivery approach. Cell survival after transplantation is compromised by several factors, including ECM detachment, mechanical stress, hypoxia, nutritional deficiency, and immune-mediated injury [20]. Furthermore, studies have shown that only a small percentage of infused MSCs are retained within target organs, with many cells becoming trapped in non-target tissues such as the lungs following intravenous administration [21,22]. Therefore, advanced technologies capable of enhancing cell homing, retention, and survival are required to improve regenerative efficacy.

Cell sheet technology has emerged as a scaffold-free and biomaterial-independent cell delivery platform that offers several advantages, including reduced foreign body reactions, enhanced cell viability, potent paracrine activity, and efficient targeting of damaged tissues [21,23,24,25,26,27]. By eliminating the need for exogenous scaffolds, cell sheet technology addresses several limitations associated with conventional single-cell transplantation and biomaterial-based approaches. Importantly, cell sheets preserve intercellular junctions, endogenous extracellular matrix (ECM) components, and cell-surface molecules, thereby promoting the retention and survival of transplanted cells at the target site. These properties facilitate not only tissue reconstruction but also therapeutic benefits mediated by paracrine signaling, immunomodulatory functions, anti-fibrotic effects, and restoration of the local tissue microenvironment.

In this review, we first summarize the biological and technological basis and clinical applications of cell sheet technology. We then define functional regeneration in relation to established regenerative concepts and discuss liver cirrhosis as a representative condition in which functional recovery may occur without complete anatomical reconstitution. Finally, we examine KCS-003 as a preclinical case study and discuss its current evidence, limitations, safety considerations, and translational challenges. In addition, clinical investigations, including corneal and retinal reconstitution, esophageal regeneration, cartilage repair, and cardiac patches, were shown as representative clinical cases of cell sheet technology.

## 2. Cell Sheet Technology as a Scaffold-Free and Biomaterial-Free Platform

Cell sheet engineering is an established tissue engineering platform based on the fabrication of ECM-rich cellular constructs that can be transplanted without exogenous scaffolds [21,23,24,25,26,27]. Central to this technology is the use of temperature-responsive culture surfaces coated with poly(N-isopropylacrylamide) (PNIPAAm), a polymer that exhibits reversible changes in surface properties depending on temperature. While hydrophobic at 37 °C, facilitating cell attachment and proliferation, PNIPAAm becomes hydrophilic at temperatures below 32 °C, leading to the gentle release of intact cell monolayers. Unlike conventional harvesting methods, this approach eliminates the need for proteolytic enzymes and thus preserves the native ECM, intercellular connections, and membrane-associated proteins. As a result, harvested cell sheets retain high viability and biological functionality, enabling efficient engraftment and improved interactions with host tissues. These advantages have contributed substantially to the advancement of regenerative medicine and tissue repair strategies.

Building on this foundation, the transition from clinically validated somatic cell sheets to mesenchymal stem cell (MSC) sheets represents a significant conceptual expansion of technology [21]. MSC therapeutic efficacies are inconsistent, largely attributed to transplanted MSC difficulties both in engrafting at tissue sites and in retaining their therapeutic functions from suspension formulations [27]. MSC functional components, including cell adhesion and cell–cell junction proteins, and the ECM that contribute to essential cellular therapeutic effects, are damaged or removed by proteolytic enzymes used in stem cell harvesting strategies from culture. Cell sheet technology permits cell harvest without cell protein damage. Retention of cellular structures including the ECM, cell–cell junctions, and cell–ECM junctions is correlated with human MSC survival after detachment from cell culture surfaces. When trypsin is used for detachment of MSCs from the dish, MSCs have been reported to lose integrin β-1 (cell–ECM junction) by trypsin treatment [27]. In addition, although Yap expression was similar among the cell sheet, trypsin treatment, and scraper-harvested groups, expression of phosphorylated Yap was increased in trypsin-treated cells compared to cell sheet and cell scraper groups, suggesting that apoptosis is induced by trypsin treatment but not cell sheet technology [27]. These data suggest that cell sheet technology is proposed to enhance stem cell survival and their function in stem cell-based therapy.

## 3. Framework of Functional Regeneration

Regenerative medicine was initially envisioned as a means of restoring tissue function through the transplantation and integration of therapeutic cells into damaged organs. Nevertheless, clinical studies have revealed significant challenges associated with this approach, particularly with respect to long-term cell survival and stable tissue integration [3]. Furthermore, the pathophysiology of chronic disorders extends beyond simple cell loss and is often characterized by persistent inflammation, excessive fibrosis, vascular dysfunction, and maladaptive remodeling [28,29]. Consequently, there is increasing recognition that successful regenerative therapies must promote an environment conducive to endogenous tissue repair in addition to providing exogenous cellular replacement.

Recent studies suggest that the therapeutic efficacy of stem cell therapies is largely attributable to their paracrine activity rather than their capacity to engraft and directly reconstruct damaged tissues [30,31]. Through the release of soluble factors and extracellular vesicles, stem cells exert multiple biological effects, including immunomodulation, anti-fibrotic activity, neovascularization, and activation of endogenous repair pathways [32,33]. This shift in understanding has redefined the role of transplanted cells, positioning them as regulators of regenerative microenvironments instead of simple cellular replacement agents. In several preclinical models, particularly those involving MSCs and cardiac-derived cells, therapeutic benefits have been attributed predominantly to secreted factors rather than durable engraftment or direct tissue replacement. However, the relative contribution of paracrine mechanisms varies according to cell type, disease model, delivery route, and outcome measure, and no single quantitative estimate can be generalized across stem cell therapies [34].

The growing recognition of paracrine regeneration has prompted a re-evaluation of the traditional paradigm of regeneration, which was largely centered on complete tissue replacement [35]. Both experimental and clinical studies have demonstrated that substantial therapeutic benefits may be obtained without achieving full anatomical reconstruction. Functional improvement is frequently associated with modulation of inflammatory responses, suppression of fibrotic remodeling, restoration of vascular homeostasis, and activation of intrinsic repair mechanisms. Collectively, these processes can lead to meaningful clinical improvement even when structural regeneration remains partial.

In this review, we use the term “functional regeneration” as an integrative therapeutic framework rather than as a newly discovered biological mechanism. It refers to the restoration or sustained improvement of clinically meaningful organ function through modification of the pathological tissue microenvironment and activation of endogenous repair processes, without requiring complete anatomical reconstitution or permanent structural engraftments of transplanted cells.

**Figure 1:**
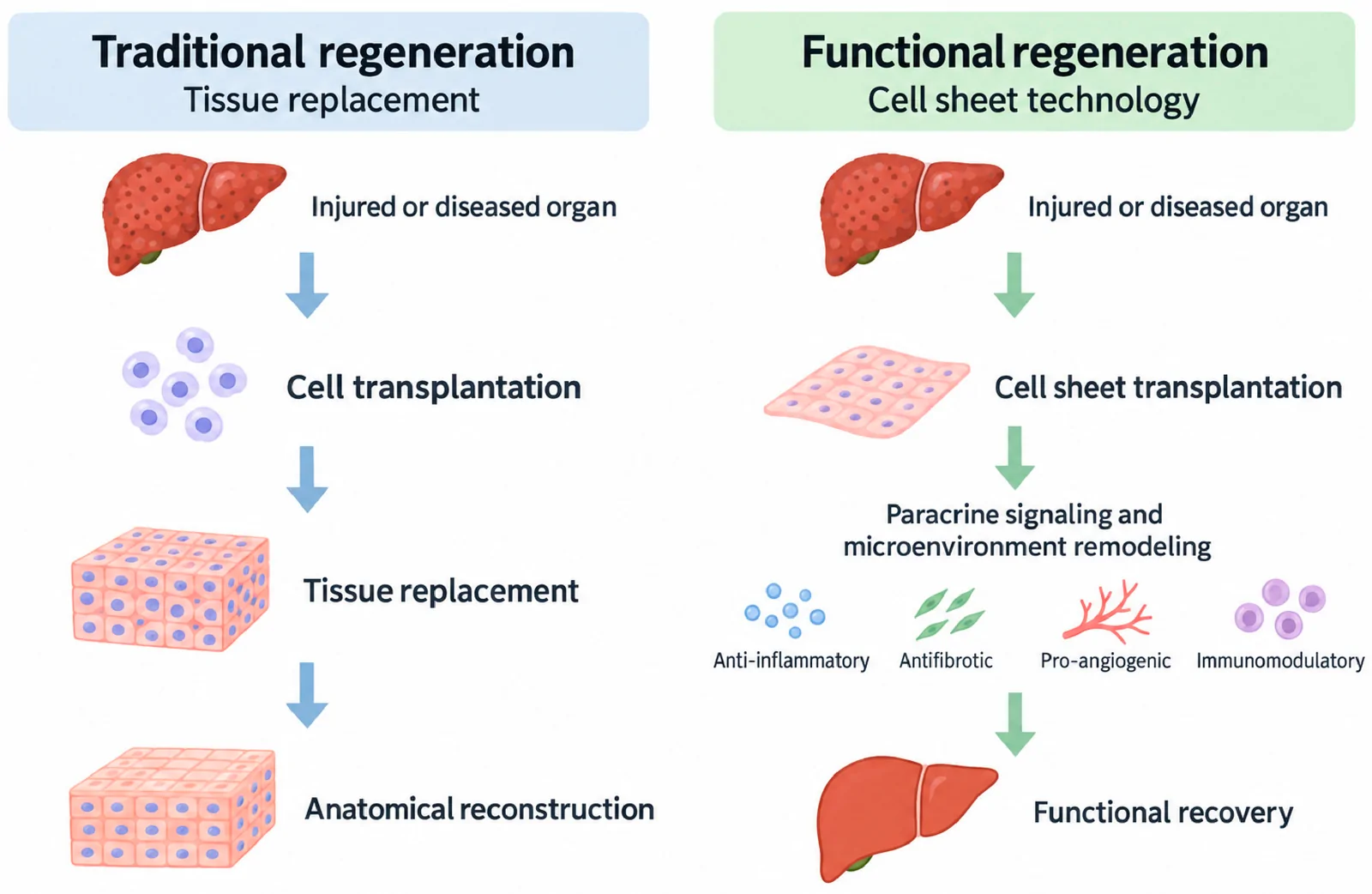
**Evolution of traditional regeneration therapy toward functional regeneration.** The left side of the figure shows traditional regeneration therapy and its outcomes, while the right side shows functional regeneration therapy and its outcomes.

Compared with the other four concepts, such as conventional regenerative medicine, tissue repair, paracrine regeneration, and disease-modifying cell therapy, conventional regenerative medicine represents the replacement of the lost cells or tissues, while a key distinction of functional regeneration is that it does not require complete tissue replacement. Tissue repair is primarily aimed at healing, closing, and stabilizing the site of injury, whereas functional regeneration emphasizes the restoration of organ function and improvement of disease progression rather than merely providing a temporary repair. Paracrine regeneration or secretome-driven regeneration is primarily defined by the promotion of tissue repair through secreted factors. Although such paracrine signaling represents a central mechanism in functional regeneration, the latter is not limited to this mechanism alone. Disease-modifying cell therapy focuses on modifying disease progression through cell-based therapy. Although it conceptually overlaps with functional regeneration, the latter places explicit emphasis on the establishment of a regenerative local microenvironment and the restoration of organ function. This framework overlaps with established concepts of paracrine regeneration, tissue repair, and disease-modifying cell therapy. Its relevance to cell sheet technology lies in the capacity of cell sheets to retain viable cells at the target site while preserving native ECM and cell–cell interactions, thereby potentially enabling spatially localized and sustained therapeutic signaling (Figure 1).

## 4. Liver Cirrhosis as a Representative Target Disease for Functional Regeneration

### 4.1. Unmet Medical Need

Liver cirrhosis is an important cause of morbidity and mortality in people with chronic liver disease worldwide [36,37,38]. Cirrhosis was associated with 2.4% of global deaths in 2019. The epidemiological data suggest that decompensated cirrhosis is increasing in prevalence. The transition from a compensated state to a decompensated state occurred at a rate of 5–12% per year. The proportion of decompensated cirrhosis associated with hepatitis B virus (HBV) and hepatitis C virus (HCV) infection, alcohol consumption, metabolic dysfunction-associated fatty liver disease (MAFLD), and other causes was 28%, 25%, 23%, 9%, and 16%, respectively, in 2017 [36]. Although compensated cirrhosis is typically clinically silent with a median survival of >12 years, decompensated cirrhosis has an expected median survival of 2 to 4 years [39]. In contrast, 5- and 10-year survival following liver transplantation (LT) is 81% and 64%, respectively. The therapeutic options, including pharmacological treatments for liver cirrhosis, primarily focus on managing complications, including portal hypertension, ascites, hepatic encephalopathy, and variceal bleeding; however, therapies capable of reversing fibrosis and restoring hepatic function remain limited despite current treatment options. LT is the only definitive treatment for decompensated cirrhosis [39]. Especially in Japan, the number of LTs performed remains insufficient because of the severe shortage of donor organs [40]. Therefore, decompensated cirrhosis represents a disease with a significant unmet medical need, as effective therapeutic options are unavailable for many patients.

### 4.2. Biological Barriers to Liver Regeneration in Liver Fibrosis/Cirrhosis

Liver regeneration is severely impaired in liver fibrosis and cirrhosis, particularly in decompensated cirrhosis, owing to multiple biological barriers. First, extensive extracellular matrix (ECM) accumulation and scar formation disrupt the normal regenerative niche, leading to architectural distortion, vascular remodeling, loss of functional parenchymal cells, and persistent inflammation. These pathological alterations create an unfavorable environment for the engraftment, survival, and regenerative activity of transplanted cells [41].

Second, chronic inflammation and immune dysregulation substantially hinder regenerative processes. Cirrhosis develops following repeated liver injury and is characterized by progressive fibrosis, hepatocellular dysfunction, and sinusoidal vascular abnormalities. Persistent activation of inflammatory cells, particularly macrophages, promotes fibrogenesis and disease progression [42,43,44]. Moreover, cirrhosis is associated with profound disturbances in immune homeostasis, including immune cell dysfunction, exhaustion, and impaired synthesis of immune mediators, resulting in persistent systemic inflammation that further compromises regenerative capacity [45].

Third, hepatocyte senescence represents another important obstacle to regeneration. Senescent hepatocytes accumulate in chronic liver diseases irrespective of etiology and show a strong association with fibrosis severity [46,47]. These cells secrete bioactive mediators that stimulate hepatic stellate cell (HSC) activation and fibrogenesis, thereby accelerating disease progression and impairing hepatic regenerative responses.

Fourth, the ductular reaction (DR), although initially considered a compensatory regenerative response, may become maladaptive in chronic liver disease. DR is activated when the regenerative potential of mature hepatocytes is insufficient and involves the expansion of hepatic progenitor cells (HPCs) derived from biliary epithelial cells (BECs) [48,49,50]. While transient DR may contribute to tissue repair, persistent and dysregulated DR has been linked to chronic inflammation, fibrogenesis, and deterioration of liver function [51,52]. Furthermore, chronic inflammatory stress can induce senescence in BECs and HPCs, leading to the development of a senescence-associated secretory phenotype (SASP) that amplifies local inflammatory and profibrotic signaling.

Fifth, advanced cirrhosis is accompanied by profound vascular remodeling, including sinusoidal capillarization, endothelial dysfunction, and portal hypertension [52]. These vascular abnormalities impair oxygen and nutrient delivery to the hepatic parenchyma, thereby limiting the regenerative potential of the liver.

Sixth, regenerative signaling pathways become dysregulated during chronic liver injury. Under physiological conditions, liver regeneration is coordinated through tightly regulated interactions among hepatocytes, liver progenitor cells, and stromal components [53,54]. However, persistent injury shifts the balance from regeneration toward fibrogenesis. Key molecular pathways involved in tissue repair, including TGF-β/Smad, Wnt/β-catenin, Hippo/YAP, and Hedgehog signaling, are also implicated in fibrotic remodeling. Whereas transient activation of these pathways supports regeneration and matrix remodeling, their sustained activation promotes pathological fibrosis. In this context, liver fibrosis may be regarded as an aberrant or maladaptive regenerative response.

Collectively, these pathological processes establish a microenvironment that is highly unfavorable for effective liver regeneration. Therefore, successful regenerative therapies for liver fibrosis and cirrhosis will require strategies capable of overcoming these biological barriers and restoring tissue function. This concept supports the emerging paradigm of functional regeneration, which focuses not only on structural tissue replacement but also on modulation of the pathological microenvironment to promote endogenous repair and improve clinical outcomes.

### 4.3. KCS-003

#### 4.3.1. KCS-003 as Cell Sheet Therapy Using IC-2-Mediated MSCs

KCS-003 is a cell sheet-based regenerative therapy that utilizes autologous bone marrow-derived MSCs treated with IC-2 prior to transplantation [55,56,57,58]. Treatments by MSCs have been effective for liver fibrosis/cirrhosis [59,60]. IC-2 is a small-molecule inhibitor of the Wnt/β-catenin signaling pathway and was developed by the authors as a derivative of ICG-001 [55]. Wnt/β-catenin signaling plays essential roles in the lineage restriction of stem cells [61]. Our previous report demonstrated that Wnt/β-catenin signaling is suppressed during hepatic differentiation of human MSCs [62,63]. Suppression of Wnt/β-catenin signaling molecules or target genes induced the hepatic differentiation of human MSCs [62]. We developed IC-2, a small-molecule compound, which suppresses the Wnt/β-catenin pathway. The induction of IC-2 has accelerated the clinical application of KCS-003. KCS-003 is produced by culturing bone marrow-derived mesenchymal stem cells on temperature-responsive culture dishes in the presence of IC-2 for 9 days, and it is then applied to the surface of the liver in patients with decompensated cirrhosis. In addition, the establishment of manufacturing under Good Gene, Cellular, and Tissue-based Products Manufacturing Practice (GTCP), lot-to-lot consistency, scalability, transport logistics, quality control, potency assays, and reproducibility challenges associated with cell sheet therapy is required. KCS-003 is discussed here as a preclinical case study of an MSC-based cell sheet designated to combine anti-fibrotic and pro-regenerative activities. Its inclusion is intended to illustrate the potential application of the functional regeneration framework to liver cirrhosis. However, its efficacy and safety have not yet been established in patients.

The key characteristics of KCS-003 are as follows:

(1) It addresses a significant unmet medical need in patients with decompensated liver cirrhosis, for whom no effective therapeutic options are currently available.
(2) It demonstrates strong therapeutic potential.
(3) It may have the potential to provide sustained biological effects: this remains to be evaluated clinically.
(4) It is supported by well-established and scientifically validated mechanisms of action.
(5) By treating the underlying liver cirrhosis, it may reduce the risk of hepatocellular carcinoma development, although whether anti-fibrotic effects of KCS-003 could influence long-term hepatocellular carcinoma risk remains unknown.
(6) It could potentially be evaluated as a supportive or bridging strategy in future clinical studies.

These characteristics are consistent with the concept of functional regeneration, which is defined as the restoration of biologically meaningful organ function through regenerative processes that modify disease pathophysiology and alter the natural history of a disease, regardless of whether complete anatomical restoration is achieved. KCS-003 is currently in preparation for clinical evaluation of the treatment of decompensated liver cirrhosis.

Hepatitis C virus (HCV) infection has been a leading cause of liver cirrhosis worldwide. Recently, an arsenal of novel and highly effective direct-acting antiviral (DAA) drugs has transformed the landscape by curing HCV in a broad range of patients, including those with established advanced fibrosis and cirrhosis. It was reported that the sustained viral response (SVR) rates of cirrhotic patients with Child-Pugh A, Child-B, and Child-Pugh C were 96.9%, 93.1%, and 83.3%, respectively [64]. Although SVR rates in patients with cirrhosis were high, hepatocellular carcinoma (HCC) development and decompensation events frequently occurred even after achieving SVR [64]. Overall, one third to two thirds of patients with HCV eradication reduced liver fibrosis after SVR in cirrhosis; however, the other one third to two thirds of the patients did not [65]. Among DAA-treated patients who achieved SVR, increased risk for hepatocellular carcinoma persists up to 10 years after HCV eradication in patients with baseline cirrhosis or high FiB-4 scores, suggesting that liver fibrosis is a risk factor for development of hepatocellular carcinoma [66]. Thus, liver cirrhosis patients with HCV require anti-fibrotic therapy even after they accomplish SVR.

#### 4.3.2. Efficacy, Molecular Mechanisms, and Safety Considerations of KCS-003

##### Acute Liver Injury Model

**Figure 2:**
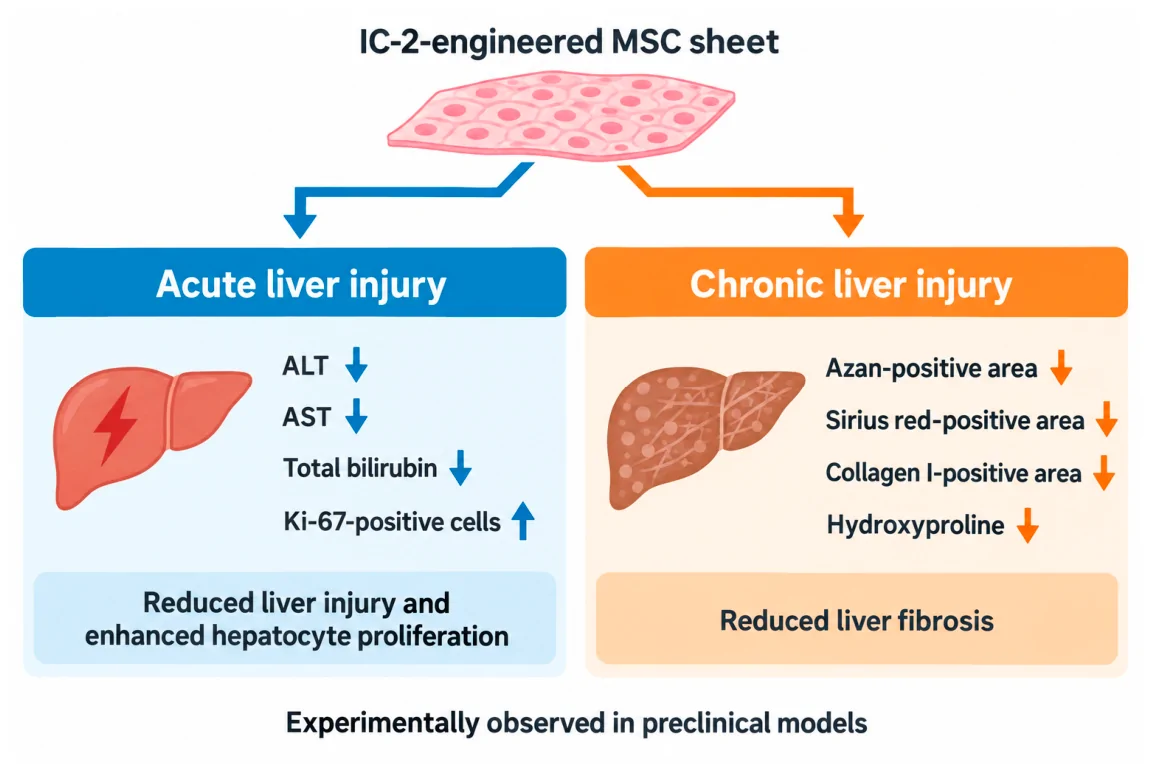
**Therapeutic effects of cell sheet transplantation using KCS-003 in acute liver injury and chronic liver injury.** The left panel illustrates the therapeutic efficacy of KCS-003 in acute liver injury, while the right panel illustrates its therapeutic efficacy in chronic liver disease. The graphs are newly prepared for the present review based on previously published data, with the corresponding references [56,57].

In the acute liver injury mouse model induced by CCl4, the levels of ALT, AST, and total bilirubin were dramatically reduced on 2 days after transplantation of KCS-003, compared to the sham group [56]. The mice transplanted with KCS-003 showed accelerated recovery of body weights. Indeed, the liver-to-body weight ratio on day 8 was also improved in mice transplanted with KCS-003, with the value being close to that of the mice that were not treated with CCl4. These data suggest that transplantation of KCS-003 is effective for acute liver injury (Figure 2).

**Figure 3:**
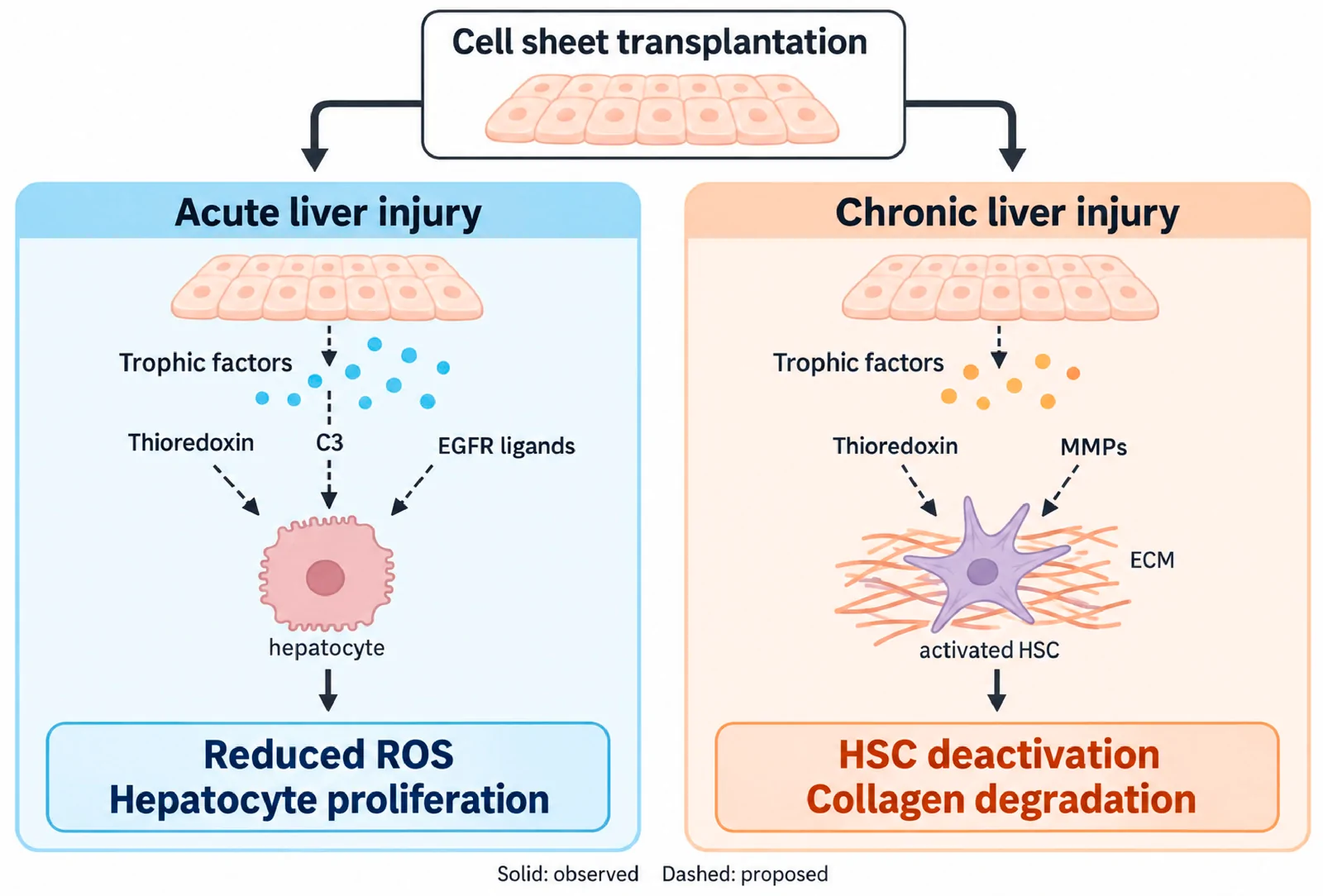
**Molecular basis of the therapeutic effects of cell sheet transplantation using KCS-003 in acute and chronic liver injury.** In acute liver injury, the trophic factors, including thioredoxin, C3, and EGFR ligands secreted from KCS-003, act on hepatocytes, resulting in a marked decrease in reactive oxidative stress (ROS) and a significant increase in hepatocyte proliferation. In chronic liver injury, the trophic factors, including thioredoxin and matrix metalloproteinases (MMPs), secreted from KCS-003, act on activated hepatic stellate cells (HSCs), resulting in marked increases in HSC deactivation and collagen degradation. Solid arrows indicate effects demonstrated in the experimental models, whereas dashed arrows indicate proposed and potential mechanisms.

In this situation, a significant increase in Ki-67-positive hepatocytes was observed on days 3 and 4 in KCS-003-transplanted mice [56] (Figure 3). Furthermore, mice transplanted with KCS-003 showed remarkable PCNA expression with a gradual increase from day 2 to day 4. These data suggest that KCS-003 possesses potent regenerative properties. Complements C3a and C5a are well known as the priming factors for liver regeneration [67]. C3 was potently expressed from day 2 to 4 in the liver transplanted with KCS-003. On day 2 after transplantation, C5a, an active form of C5, was upregulated in the livers transplanted with KCS-003. Since C5a plays a crucial role in liver regeneration through activating NF-κB and STAT3, the activation of NF-κB and STAT3 in the liver transplanted with KCS-003 was examined. The nuclear expression of NF-κB was significantly higher in mice transplanted with KCS-003 compared to the sham-operated mice. A prominent increment of phosphorylated STAT3 was observed in the livers transplanted with KCS-003 two days after transplantation. These data suggest that IC-2-mediated complement C3 activated the STAT3 pathway via C5a-mediated nuclear translocation of NF-κB in the liver transplanted with KCS-003 [56].

EGFR receptor ligands, such as HB-EGF and amphiregulin, were increased in the liver transplanted with KCS-003. Especially, amphiregulin was potently expressed, followed by higher expression of phosphorylated EGFR, suggesting that HB-EGF and amphiregulin play an important role in the acceleration of liver regeneration in this model.

The reductive effect on oxidative stress was also examined since CCl4 is metabolized by cytochrome p450 enzymes to a highly reactive trichloromethyl radical, which generates oxidative stress. The 8-OHdG-positive cells were observed in the livers of the sham-operated mice on day 2 and day 3 after transplantation; however, these were reduced in the liver transplanted with KCS-003. On day 2 after transplantation, prominent expression of thioredoxin (TRX) and thioredoxin reductase 1 (TRXR1) was observed in the liver transplanted with KCS-003 [56], suggesting that the TRX system potently contributes to the reduction of oxidative stress.

The survival rates of the mice, which received cell sheet therapy treated with hexachlorophene, a Wnt/β-catenin signal inhibitor, were significantly improved compared to sham-operated mice [68], suggesting that cell sheet technology using a Wnt/β-catenin inhibitor modifies disease pathophysiology and may alter the natural history of a disease.

##### Chronic Liver Injury Model

In the chronic liver injury model caused by repetitive administration of CCl4, serum ALT was significantly decreased by KCS-003; however, serum AST and total bilirubin were not changed at the time of sacrifice [57]. The histochemical analysis, including Azan staining, Sirius red staining, and immunohistochemistry for type I collagen, showed a significant reduction in livers transplanted with KCS-003. Moreover, hydroxyproline contents in liver tissues were also reduced in KCS-003-treated livers (Figure 2). Further, both type I collagen and type III collagen were decreased; especially, type I collagen was prominently reduced in western blot analysis. These data suggest that KCS-003 significantly reduced liver fibrosis.

In chronic liver injury, the therapeutic mechanisms were evaluated (Figure 3). Liver fibrosis is characterized by the excessive accumulation of ECM as a result of a disruption in the balance between fibrogenesis and fibrolysis. Fibrogenesis is regulated by activated hepatic stellate cells (HSCs). The expression of alpha-smooth muscle actin (α-SMA), a major indicator of HSC activation, was markedly decreased in the liver treated with KCS-003. Upregulation of TRX protein by IC-2 suggests that TRX really suppresses HSC activation [63]. On the other hand, since the matrix metalloproteinases (MMPs) that are involved in the degradation of type I collagen are MMP-1, MMP-2, MMP-13, and MMP-14, expression of these MMPs in response to IC-2 was examined. IC-2 upregulated the mRNA expression of MMP-1, MMP-2, and MMP-14 in MSCs in vitro. Protein expression of MMP-1, MMP-2, and MMP-14 was increased by IC-2, and the active form of MMP-13 was also increased. Since hepatic hydroxyproline contents on the ninth day after transplantation were inversely correlated with relative MMP-1 and MMP-14 activity before transplantation, knockdown experiments of MMP-1 and MMP-14 through small interfering RNA (siRNA) transfection during the preparation of KCS-003 were performed. As a result, transplantation of si-MMP-1-transfected IC-2 sheets achieved almost the same reduction in liver fibrosis as si-Ctrl-transfected IC-2 sheets. However, the anti-fibrotic effect was diminished with IC-2 sheets transfected with si-MMP-14, suggesting that the fibrolytic activity of KCS-003 on liver fibrosis is mainly dependent on MMP-14 [57]. The univariate analysis between hepatic hydroxyproline and cell sheet-secreted factors such as MMP-1, MMP-2 activity, MMP-13 activity, MMP-14, thioredoxin, amphiregulin, and C3 in cell sheets indicated that secreted MMP-14 is inversely correlated with hepatic hydroxyproline content [58]. Furthermore, the univariate analysis between hepatic hydroxyproline and factors such as MMP-1 activity, MMP-2 activity, MMP-13 activity, MMP-14 activity, and thioredoxin in cell lysates indicated that MMP-13 activity and thioredoxin are inversely correlated with hepatic hydroxyproline content, respectively. These data suggest that KCS-003 suppresses liver fibrosis through inhibition of activated HSCs and induction of MMPs. Especially, secreted MMP-14 plays an important role in the anti-fibrotic effect of KCS-003 [58].

The translational relevance of the acute and chronic CCl4 models should be interpreted differently. The acute liver injury model primarily reflects hepatocellular damage and regenerative responses following acute toxic injury and is useful for evaluating hepatoprotective and regenerative effects. In contrast, the chronic CCl4 model mimics certain aspects of progressive liver fibrosis and is widely used for assessing anti-fibrotic therapies. However, the chronic CCl4 model has important limitations as a representation of human cirrhosis. Human cirrhosis develops over many years and arises from diverse etiologies, including viral hepatitis, alcohol-associated liver disease, and metabolic dysfunction-associated steatotic liver disease. Furthermore, the CCl4 model does not fully reproduce the architectural complexity, portal hypertension, immune dysregulation, systemic complications, and clinical heterogeneity characteristic of advanced human cirrhosis. Accordingly, the findings from the CCl4 model should be regarded as proof-of-concept evidence supporting the biological activity of KCS-003 rather than definitive evidence of clinical efficacy in human cirrhosis.

##### Safety Considerations for KCS-003

The safety profile of KCS-003 is supported by several characteristics of mesenchymal stromal cell (MSC)-based cell sheet technology. Cell phenotype stability is maintained through controlled manufacturing processes and quality control testing. Cells consistently expressed CD90 and CD105 while lacking hematopoietic markers, such as CD34 and CD49. The risk of unwanted differentiation is considered low because MSCs are transplanted as a cell sheet without prior induction toward non-target lineages. No evidence of tumor formation was observed in preclinical studies, as MSCs exhibit limited proliferative capacity and have not been associated with malignant transformation in clinical applications. Immunologically, MSCs are known to exert immunomodulatory effects and generally display low immunogenicity, although immune responses should continue to be monitored in future clinical studies. Long-term persistence of transplanted cells appears limited, with accumulating evidence suggesting that therapeutic efficacy is mediated predominantly through paracrine mechanisms rather than permanent engraftment. In addition, manufacturing consistency is ensured through standardized cell sourcing, culture conditions, release criteria, and quality control assessments, which are essential for clinical translation and regulatory compliance.

## 5. Clinical Applications of Cell Sheet Technology to Other Organs Systems

The clinical potential of this platform has been evaluated in several human studies (Table 1). Nishida et al. reported the transplantation of autologous oral mucosal epithelial cell sheets for the treatment of bilateral total corneal stem cell deficiencies [69]. In this disease, if corneal epithelial stem cells are completely absent and the sources of corneal epithelial cells are exhausted, the peripheral conjunctival epithelium invades inwardly, and the corneal surface becomes enveloped by vascularized conjunctival scar, resulting in corneal opacification that leads to severe visual impairment. In this report, there were 3-by-3 mm specimens of oral mucosal tissue from four patients with bilateral total corneal stem cell deficiencies. Tissue-engineered epithelial cell sheets were fabricated ex vivo by culturing harvested cells for two weeks on temperature-responsive cell culture surfaces. After surgical removal of conjunctival fibrovascular tissue, cell sheets were transplanted directly to the denuded corneal surfaces. Complete re-epithelialization of the corneal surfaces occurred within one week in all four patients. As a result, corneal transparency was restored and postoperative visual acuity remarkably improved. During a mean follow-up of 14 months, all corneal surfaces remained transparent. No complications were observed. The authors concluded that structureless transplantation of cell sheets may be used to reconstruct corneal surfaces and can restore vision in patients with bilateral total corneal stem cell deficiencies.

Ohki et al. reported endoscopic transplantation of cultured autologous oral mucosal epithelial sheets to esophageal ulcers after endoscopic submucosal dissection (ESD) [70]. Esophageal stricture is the major complication after ESD. It was reported that mucosal removal of more than 3/4 of the luminal circumference was associated with a high rate of stricture in patients with esophageal squamous cell carcinoma and that mucosal defects longer than 30 mm were associated with greater stricture severity. To prevent esophageal stricture, the authors started clinical research on the endoscopic transplantation of autologous oral mucosal epithelial cell sheets. Clinical research was performed at Tokyo Women’s Medical University, Nagasaki University, and Karolinska Institute. Of 25 patients who received this therapy, esophageal stricture was prevented in 17 patients. The duration of wound healing was 2 to 70 weeks. Since the submucosal layer is rich in blood vessels for blood supply, transplantation of the cell sheets is physically beneficial on the dissected surface where fibrin meshes cannot adhere. No adverse effects were observed. The authors concluded that cell sheet technology has been proven to be a highly safe regenerative therapy.

Sato et al. reported that after conventional surgical treatment for osteoarthritis of the knee (OAK), chondrocyte cell sheet transplantation improved clinical and structural outcomes in eight patients [71]. Osteoarthritis (OA) is the most prevalent form of musculoskeletal disease worldwide and is the most common cause of mobility loss that adversely affects quality of life, work productivity, and cost of health care. The authors reported the combination therapy in which conventional surgical treatment for OAK is followed by autologous transplantation of chondrocyte sheets for cartilage repair. The combination therapy is effective, as assessed by MRI, arthroscopy, viscoelasticity, histology, and clinical outcomes KOS and LKS. The follow-up periods are 36 to 61 months. There were 26 adverse events reported, including pain, leukocytosis, increased C-reactive protein level, swelling, and fever. However, the authors considered that these events were likely to have been related to the high tibial osteotomy or anterior cruciate ligament reconstruction, but not transplantation of chondrocyte sheets. The authors conclude that therapy requires longer-term follow-up and strict comparative studies.

Mandai et al. reported that after the removal of the neovascular membrane, there was a transplantation of the autologous-induced pluripotent stem cell (iPSC)-derived retinal pigment epithelial (RPE) cell sheet under the retina in a patient with age-related macular degeneration (AMD) [72]. AMD is the most prevalent retinal disease that threatens vision in older people in developed countries. A high rate of recurrence was seen in current treatments, including intravitreal injection of anti-vascular endothelial growth factor (VEGF), and earlier treatments, such as surgical removal of the neovascular membrane, caused some problems, such as atrophy of the retina and choroid. The iPSCs were generated from skin fibroblasts from two patients and were differentiated into retinal pigment epithelial (RPE) cells. After removal of the neovascular membrane, the transplantation of the autologous iPSC-derived RPE cell sheet was performed in one of the patients. At 1 year after surgery, the transplanted cell sheet remained intact, best corrected visual acuity had not improved or worsened, and cytoid macular edema was present. There were no serious complications. In the other patients, single-nucleotide variations previously reported to be cancer driver mutations were found, and then the transplantation was not performed.

Kawamura et al. reported that iPSC-derived cardiomyocyte patch transplantation was performed in three patients with ischemic cardiomyopathy [73]. Ischemic cardiomyopathy is increasing in number worldwide with aging. Ischemic cardiomyopathy generally worsens from the onset of heart failure symptoms, and remission and exacerbation repeatedly occur. The inclusion criteria of the patients were a left ventricular ejection fraction of 35% or less and heart failure symptoms of the New York Heart Association class III or higher despite existing therapies such as revascularization. Three patients received iPSC-derived cardiomyocyte patch transplantation, and they were observed for a year. In the first case, no transplanted cell-related adverse events were observed, and heart failure symptoms were improved. Left ventricular contractility and myocardial blood flow were improved in two patients. Four severe adverse events were observed. The authors concluded that transplantation of iPSC-derived cardiomyocyte patches for ischemic cardiomyopathy can be safely performed.

These representative organ systems, including corneal and retinal reconstitution, esophageal regenerative therapy, cartilage repair, and cardiac patches, are incorporated to truly substantiate cell sheet technology as a versatile platform for functional regeneration across multiple organs (Table 1).

## 6. Future Perspectives

Although this review has primarily focused on cell sheet technology as a platform for functional regeneration, several other emerging regenerative approaches also hold considerable promise (Table 2). One notable example is organoid transplantation. Organoids are three-dimensional, self-organizing cellular constructs that recapitulate key structural and functional features of native tissues. While most organoid-based applications remain at the preclinical stage, they are increasingly being utilized for disease modeling, drug discovery, and personalized medicine. The accumulation of encouraging preclinical data has accelerated their translation toward clinical applications [74].

Another rapidly advancing field is the development of bioartificial organs. Recent progress in cell biology, biomaterials, and bioengineering has enabled the creation of increasingly sophisticated organ replacement systems. In the liver field, a variety of bioartificial liver platforms have been developed, including extracorporeal hollow fiber bioreactors, cell encapsulation technologies, scaffold-based constructs, and implantable tissue-engineered devices designed to support or restore hepatic function [75].

Three-dimensional (3D) bioprinting also represents a promising strategy for regenerative medicine. This technology employs bioinks composed of living cells, biomaterials, and bioactive components to fabricate complex tissue-like structures with precise architectural organization. Continued advances in printing technologies, biomaterials, and cellular engineering are steadily expanding the potential clinical applications of 3D bioprinting [76,77].

In addition, small extracellular vesicles (sEVs), particularly exosomes, have attracted considerable attention as cell-free therapeutic agents. These vesicles mediate intercellular communication by delivering proteins, lipids, and nucleic acids to recipient cells, thereby modulating a variety of regenerative pathways. Although their clinical application is currently limited by challenges related to large-scale production, isolation efficiency, biological stability, and tissue-specific targeting, exosome-based therapies are expected to become an important component of future regenerative medicine strategies [78,79,80,81,82].

For cell sheet technology, several key challenges remain to be addressed before broader clinical implementation can be achieved. These include the establishment of reliable allogeneic transplantation strategies, optimization of cryopreservation methods, and the development of efficient delivery devices and transplantation systems [26,83,84,85,86,87,88,89,90,91,92,93,94,95,96,97]. Overcoming these limitations, together with advances in complementary regenerative technologies, may further enhance the therapeutic potential of functional regeneration and contribute to improved outcomes for patients with chronic and otherwise irreversible diseases. These approaches should be regarded as complementary rather than mutually exclusive.

## Figures and Tables

**Table 1:** Clinical applications of cell sheet technology.

Indication	Cell Source	Autologous or Allogeneic	Sheet Fabrication	Study Design	Patient No.	Follow-Up Periods	Main Outcome	Safety	Reference
Corneal stem cell deficiency	Oral mucosal epithelial cells	Autologous	Temperature-responsive culture	Clinical study	4	14 months	Re-epithelialization	No complications	Nishida, et al. [69]
Esophageal ulcer after ESD	Oral mucosal epithelial cells	Autologous	Temperature-responsive culture	Clinical study	25	2~70 weeks	Stricture-related outcomes	Some adverse effects	Ohki, et al. [70]
Knee osteoarthritis	Chondrocytes	Autologous	Temperature-responsive culture	Clinical study	8	36~61 months	Clinical and structural outcomes	Safety	Sato, et al. [71]
Age-related macular degeneration	iPS-derived RPE	Autologous	RPE sheet	First-in-human case	1	Over 1 year	Graft survival visual outcome	Cystoid macular edema was present	Mandai, et al. [72]
Ischemic cardiomyopathy	iPS-derived cardiomayocytes	Allogeneic	Cardiomayocyte patch	Early clinical cases	3	1 year	Perfusion and contractility	No sheet-related serious events	Kawamura, et al. [73]

**Table 2:** Comparison of cell sheet technology with other platforms.

Platform	Major Advantages	Major Limitations	Differences from Cell Sheet Technology
Cell sheet technology	Local retention Preservation of ECM and cell–cell interactions Scaffold-free	Procedure Scalability Storage Mechanical strength	
Organoids	Reproduction of tissue architecture and cellular diversity	Vascularization Functional maturity Cell viability Structural complexity	Strong potential for advanced tissue reconstitution Challenges remain in graft integration
Extracellular vesicles	Cell-free Potential for off-the-shelf use	Potency Manufacturing Biodistribution	Duration of effect remains unclear
Bioartificial organs	Support of organ function ex vivo or through medical devices	Large-scale equipment requirements Cost Long-term maintenance	Focus more on functional replacement than tissue regeneration
3D bioprinting	Customizable spatial architecture	Vascularization Functional maturity Scalability	High structural fidelity Manufacturing remains complex

## Data Availability

No new data were created or analyzed in this study. Data sharing is not applicable to this article.
